# A multidisciplinary consensus on the morphological and functional responses to immunotherapy treatment

**DOI:** 10.1007/s12094-020-02442-3

**Published:** 2020-07-04

**Authors:** L. Leon-Mateos, M. J. Garcia-Velloso, R. García-Figueiras, J. F. Rodriguez-Moreno, J. L. Vercher-Conejero, M. Sánchez, J. L. Perez Gracia, M. Simo-Perdigo, L. Gorospe

**Affiliations:** 1 Medical Oncology Department, Instituto de Investigación Sanitaria de Santiago de Compostela (IDIS), Complexo Hospitalario Universitario de Santiago de Compostela (CHUS), Servicio Galego de Saude, Santiago de Compostela, Spain; 2 Nuclear Medicine Department, Clinica Universidad de Navarra, Av. Pio XII 36, 31008 Pamplona, Spain; 3 IdisNA, Instituto de Investigación Sanitaria de Navarra, Pamplona, Spain; 4 Radiology Department, Hospital Clínico Universitario de Santiago de Compostela, Santiago de Compostela, Spain; 5 Medical Oncology Department, Centro Integral Oncologico HM Clara Campal, Madrid, Spain; 6 Nuclear Medicine Department – PET Unit, Bellvitge University Hospital – IDIBELL, Barcelona, Spain; 7 Radiology Department, CDIC, Hospital Clínic, Barcelona, Spain; 8 Oncology Department, Clinica Universidad de Navarra, Pamplona, Spain; 9 Nuclear Medicine Department, Hospital Universitari Vall Hebron, Barcelona, Spain; 10 Radiology Department, Ramón y Cajal University Hospital, Madrid, Spain

**Keywords:** Immunotherapy, Response, Imaging, Biomarker, Immune-related adverse events

## Abstract

The implementation of immunotherapy has radically changed the treatment of oncological patients. Currently, immunotherapy is indicated in the treatment of patients with head and neck tumors, melanoma, lung cancer, bladder tumors, colon cancer, cervical cancer, breast cancer, Merkel cell carcinoma, liver cancer, leukemia and lymphomas. However, its efficacy is restricted to a limited number of cases. The challenge is, therefore, to identify which subset of patients would benefit from immunotherapy. To this end, the establishment of immunotherapy response criteria and predictive and prognostic biomarkers is of paramount interest. In this report, a group of experts of the Spanish Society of Medical Oncology (SEOM), the Spanish Society of Medical Radiology (SERAM), and Spanish Society of Nuclear Medicine and Molecular Imaging (SEMNIM) provide an up-to-date review and a consensus guide on these issues.

## Immunotherapy clinical application

**Fig. 1:**
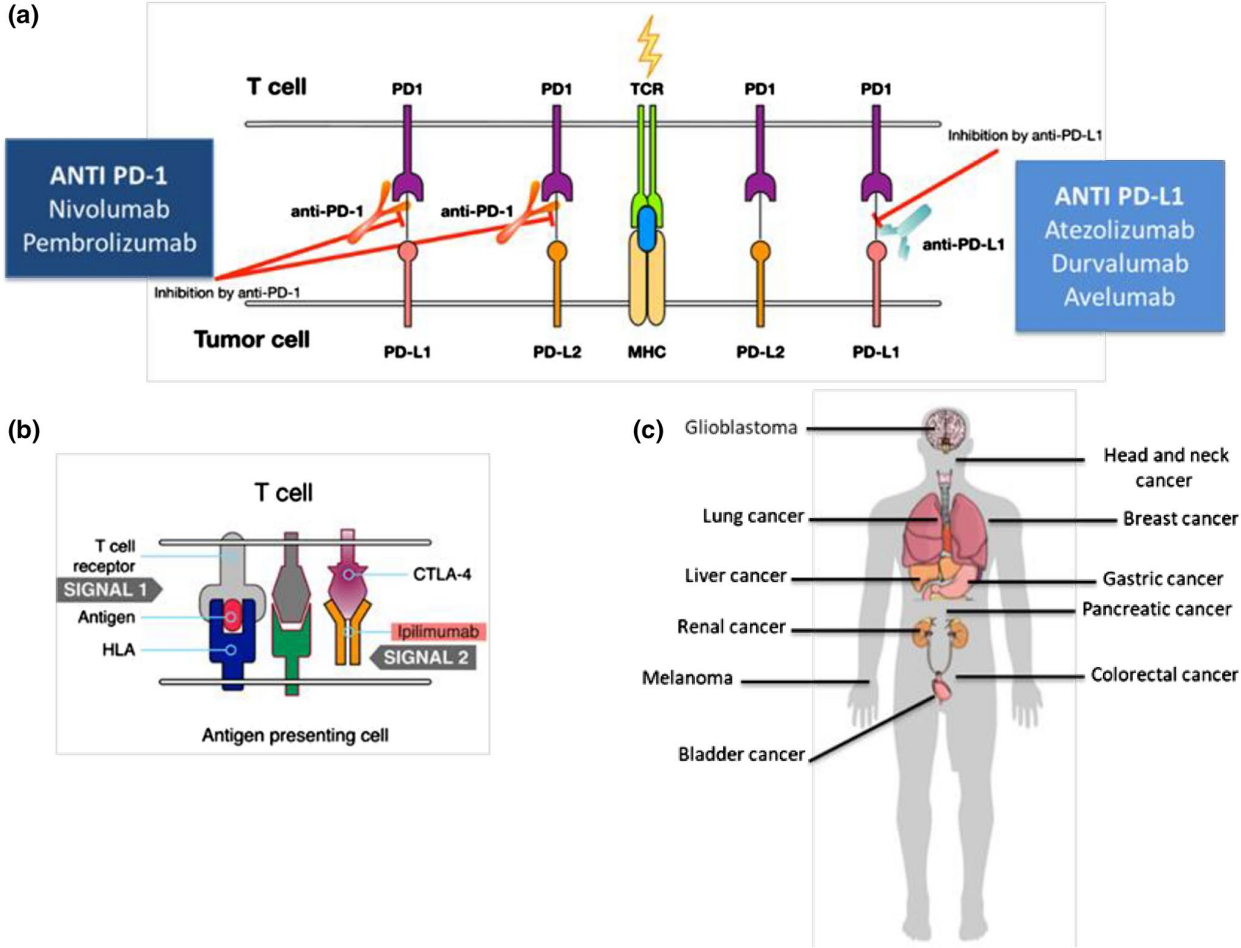
Mechanism of action of immunotherapy and main general indications. **a** The T lymphocyte recognizes the antigen presented by the major histocompatibility complex (MHC) of the tumor cell. The ligands PD-L1 and PD-L2 function as negative regulators of the immune response by binding to the lymphocyte receptor PD-1. The anti-PD-1 and anti-PD-L1 antibodies interfere with this binding, potentiating the immune response against the tumor. **b** CTLA-4 is another receptor that inhibits the action of T lymphocytes. The monoclonal antibody ipilimumab blocks CTLA-4 and potentiates the activity of T lymphocytes. **c** Several modalities of immunotherapy are approved or in development for use in different tumor locations

The implementation of immunotherapy has radically changed the treatment of oncological patients. Unlike other oncological treatments (such as chemotherapy, tyrosine kinase inhibitors and others that act directly upon tumor cells), immunotherapy exerts antitumor action by activating the patient’s immunologic response against cancer and can be used as a monotherapy or in combination with other immunotherapeutic agents, chemotherapy or targeted therapies [1–6]. Currently, immunotherapy is approved or under development for the treatment of patients with head and neck tumors, melanoma, lung cancer, bladder tumors, colon cancer, cervical cancer, breast cancer, Merkel cell carcinoma, liver cancer, leukemia and lymphomas (Fig. 1). However, its efficacy is restricted to a limited number of cases. The challenge is, therefore, to identify which subset of patients would benefit from immunotherapy [7]. There are also other important areas of research, such as the establishment of treatment response criteria according to drug mechanisms of action and identifying and managing immune-mediated toxicities.

A group of experts from the Spanish Society of Medical Oncology (SEOM), the Spanish Society of Medical Radiology (SERAM), and Spanish Society of Nuclear Medicine and Molecular Imaging (SEMNIM), selected and supported by these scientific societies, met to discuss and provide an up-to-date introductory review on some general issues on immunotherapy, and a multidisciplinary consensus on the immunotherapy response criteria and the predictive and prognostic biomarkers. In addition, functional and molecular imaging advances for immunotherapy response assessment are also reviewed. The consensus was initiated with a face-to-face meeting, where the content, procedures and topic distribution among the experts were made. The first draft of the consensus was distributed among all participants who made comments and amendments to the document that were discussed via e-mail and teleconference. The coordinators of the consensus agreed a second draft based on the comments received. This process was repeated twice until a final draft was circulated, agreed and approved by all authors.

## Immunotherapy types and mechanisms of action

Immunotherapy is a general term comprising several strategies for triggering the host immune response, including the following:

1. Checkpoint inhibitors. The immune response involves complex signal balances between T lymphocyte cytotoxic activation and inhibition [8]. Checkpoint inhibitors induce a positive balance that favors the activation of effector T lymphocytes.
2. Cytokines. Cytokines (interferon α/β, interleukin 12, etc.) facilitate adequate lymphocyte activation. Although their clinical use is limited by their marked toxicity, some cytokines, such as interleukin 2 or alpha 2b interferon, have shown results for treating melanoma and renal cell carcinoma [9].
3. Innate immunity activation. Nonspecific initial immune response activation might also facilitate cancer progression management. One example could be adjuvant treatment of urothelial carcinoma with Calmette-Guérin bacillus (BCG) [10].
4. Cell therapy. Different strategies consist of manipulating patient cells to restore their capacity to recognize and kill tumor cells.
  4.1 Tumor-Infiltrating Lymphocytes (TILs): TIL isolates are taken from tumor biopsies and replicated in ex vivo cell culture for their later infusion in patients previously submitted to myeloablative treatments [11].
  4.2 Chimeric antigen receptor (CAR) - T cells: patient’s T lymphocytes are extracted by leukapheresis and genetically modified ex vivo to recognize a given preselected antigen. Such therapy has shown remarkable activity against hematological neoplasms [12].
5. Oncolytic viruses. human viruses have been genetically modified to infect and kill tumor cells, creating a proinflammatory microenvironment triggering additional adaptive immune mechanisms [13].
6. Vaccines. specific antitumor response activation can be elicited by active immunization (vaccine administration) derived from cancer cell expressions of certain tumor-specific antigens.

## Unusual response patterns associated with immunotherapy

Tumor response to oncologic treatments has been associated with tumor size reduction. However, immunotherapy may usually cause several atypical response patterns, such pseudoprogression, hyperprogression, and dissociated responses, that are difficult to evaluate according to classic tumor response criteria and with conventional imaging techniques. Besides, immunotherapy may also achieve durable responses in a subset of patients with advanced cancer that can be maintained even after stopping treatment.

### Sustained responses

Sustained responses are one of the main response patterns associated with immunotherapy. A major breakthrough of immunotherapy is its potential to achieve lasting responses in a subset of patients with advanced cancer that can be maintained even after stopping treatment. There is no standard definition of sustained response in the literature. This kind of response appears in all cancer types, and there are current investigations underway to identify predictive factors of this feature [14–16].

### Pseudoprogression

**Fig. 2:**
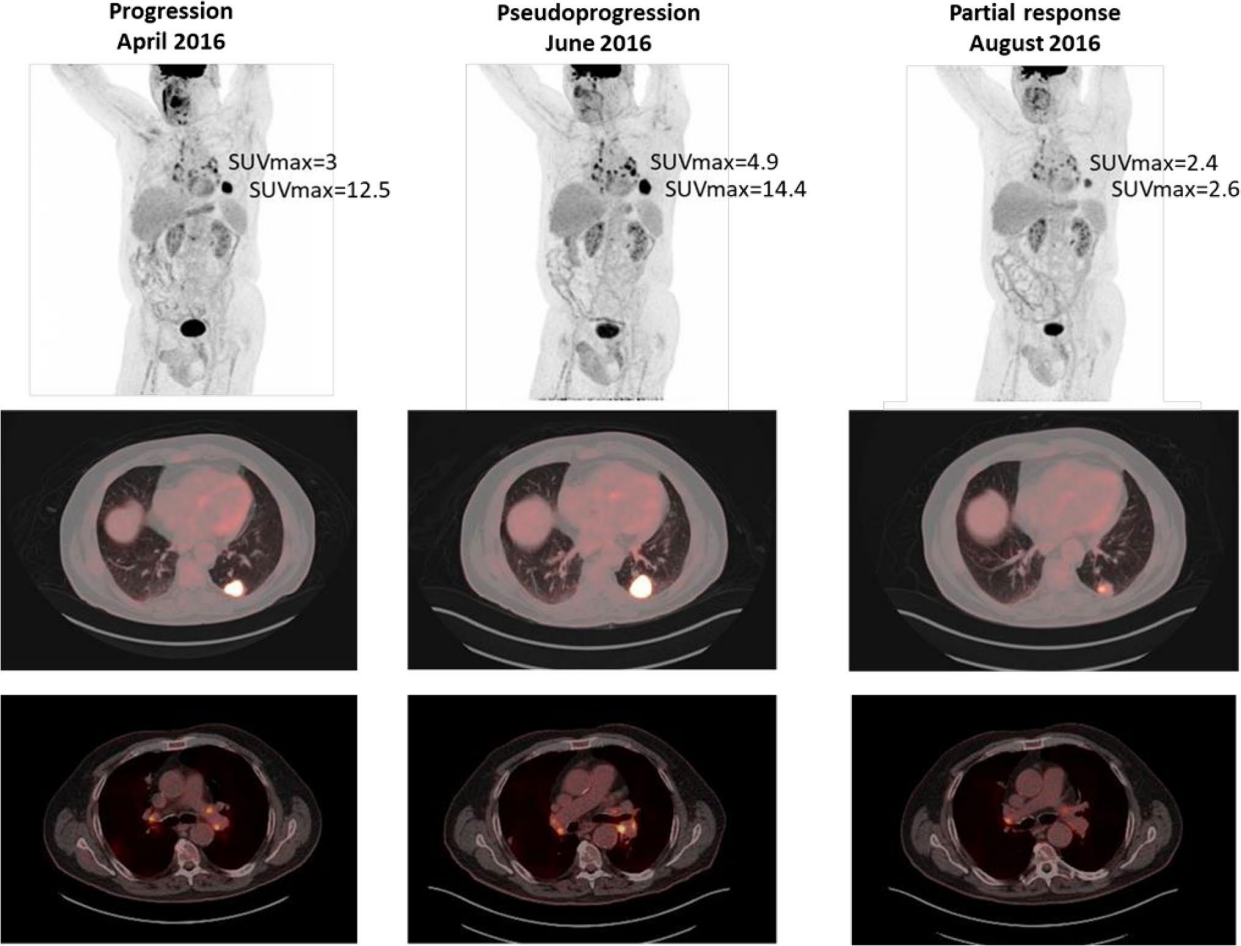
Pesudoprogression in a patient with lung adenocarcinoma. Patient diagnosed in January 2014 of well-differentiated lung adenocarcinoma T4N3M0 stage, and treated with cisplatin and pemetrexed. Progression resistant to a second-line chemotherapy was detected in April 2016. ^18^F-FDG PET/CT (**a**) identified a lesion located in LII (SUVmax of 12.5) and mediastinal and hilar lymph node uptake (SUVmax of 3). Nivolumab was started and an^18^F-FDG PET/CT study 2016 was performed for response assessment in June (**b**) revealing an increase in the metabolism and size of the lung lesion (SUVmax of 14.4) and in the number and metabolism of thoracic lymph nodes (SUVmax of 4.9). Because of clinical stability, immunotherapy was maintained and^18^F-FDG PET/CT performed in August 2016 (**c**) showed a metabolic reduction of lung lesion (SUVmax of 2.4) and lymph nodes (SUVmax of 2.6), consistent with partial response. Given the evolution, the ^18^F-FDG PET/CT(B) was interpreted as pseudoprogression

Tumor shrinkage has been identified after an initial increase in tumor burden or the appearance of new lesions. This phenomenon, called pseudoprogression, is the consequence of treatment-activated immune cells infiltrating the tumor milieu. It is associated with edema or necrosis that may cause a radiographic increase in tumor volume, including the appearance of lesions not recognized on previous imaging [17, 18]. Pseudoprogression is the most characteristic atypical response pattern associated with immunotherapy. It was first reported in melanoma patients treated with anti-CTLA4, but this response pattern may also appear with other immunotherapy agents and in different tumor types (Fig. 2) [19–21]. Pseudoprogression can appear early during treatment (within the first 12 weeks of therapy) but can also be a late phenomenon. A recent study in melanoma patients showed an early pseudoprogression rate of 4.6% and a late pseudoprogression rate of 2.8% [20]. There is a variable pseudoprogression rate among different tumor types. The rate is higher in the case of melanoma (up to 10% of patients) [22] and lower in epithelial malignancies such as lung cancer and urothelial carcinoma (1.5–3.0%). In this setting, we should consider that most of the cases of possible pseudoprogression reflect real progression. However, there is a need to establish specific response criteria for immunotherapy allowing for the continuation of treatment after apparent progression, according to classic criteria in clinically fit patients without associated toxicity.

### Hyperprogression

Hyperprogression is defined as tumor growth after the initiation of immunotherapy that is faster than under previous treatment and has been described based on retrospective observations [23]. The rate of hyperprogression is variable, ranging from 4 to 29% for different agents and tumor types [24]. Hyperprogression is prone to controversies and shows considerable incidence variability due to clinical trial heterogeneity. It is difficult to establish a difference between hyperprogression as an independent entity and rapid progression already observed in oncologic patients. Different papers published have defined multiple parameters to assess and define hyperprogression, such as the “tumor growth rate (TGR)” and the “time-to-treatment failure (TTF)”. These parameters usually combine Response Evaluation Criteria in Solid Tumors (RECIST) and elapsed time between assessments [25].

Hyperprogression correlates with worse patient survival rates. Several studies have been conducted to identify hyperprogression risk factors, including advanced age, multiple metastatic lesions, and previous radiotherapy [23, 26, 27].

### Dissociated response

A dissociated response is considered when there is regression in some target lesions but progression in others. In this scenario, it would be important to identify patients with oligoprogression, due to the possibility of treating these lesions locally. This pattern has already been described in the context of conventional chemotherapy and especially in the case of targeted therapies. A study including lung cancer patients treated with immunotherapy described a 7% rate of dissociated response [21]. In this study, patients with pseudoprogression or dissociated response showed better survival.

### Key points and recommendations

Novel patterns of response and progression to immunotherapy, which may be more frequent in immunotherapy than in chemotherapy or targeted therapies, should be known.

## Immune-related adverse events

Apart from these atypical response patterns, immune-related toxicities may appear during treatment due to self-immunity deregulation or induction. The most common adverse event is dermatologic toxicity, but immune-related toxicity could affect any organ and cause different clinical and radiological manifestations that might mimic tumor progression (Table 1) [28]. These adverse events must be identified to initiate immunosuppression treatment. Pneumonitis is the most common thoracic complication, with different radiologic manifestations [29]. A nodular pattern of organized pneumonia or sarcoidosis/sarcoid reaction could be confounded with disease progression. To differentiate these adverse events from progression, it is important to ascertain whether there is only thoracic progression and to compare the initial tumor radiological characteristics with the current tumor manifestations [30]. Other toxicities with doubtful radiologic or metabolic manifestations that could be confounded with progression are pituitary gland disorders, thyroiditis, pericarditis, colitis and pancreatitis, all characterized by volume augmentation of the different organs and increased [18F]fluorodeoxyglucose (^18^F-FDG) uptake in positron emission tomography (PET) [31, 32].

**Table 1:** Main immune-related adverse events in patients treated with immunotherapy that could interfere with the response assessment

Pulmonary	Pneumonitis Sarcoidosis/sarcoid reaction
Gastrointestinal	Colitis Hepatitis Pancreatitis
Endocrine	Primary hypothyroidism Hyperthyroidism Primary adrenal insufficiency Hypophysitis
Cardiovascular	Myocarditis Pericarditis Vasculitis Venous thromboembolism

### Key points and recommendations

- The main clinical and imaging manifestations of immune-related toxicities must be known to make an early diagnosis and to initiate early treatment of these adverse events.
- FDG-PET is a non-invasive imaging technique that allows precise localization of all potential immune-related adverse effects and to monitor the response to immunosuppression treatment.

## Immunotherapy response criteria

Tumor therapy response is categorized into four main groups: complete response [CR], partial response [PR], progressive disease [PD], and stable disease [SD]. During the last decades, several radiologic and metabolic response criteria for different oncological treatments have been described [33]. The appearance of new therapeutic strategies in recent years (targeted therapies, antiangiogenic agents and, more specifically, immunotherapy) has revealed some limitations of these criteria, such as the possibility of not detecting atypical response patterns (such as pseudoprogression) [34]. In this section, we will review the different imaging criteria described for the assessment of immunotherapy response.

### Anatomic response criteria

All radiologic response criteria for immunotherapy share common aspects, such as being based on target lesion dimension changes (measurable lesions, most representative of total tumor burden) and nontarget lesions (all the other measurable and unmeasurable lesions) defined in baseline studies. In short, differences among several response criteria to assess immunotherapy treatment are based on measurement methods (single, if based on RECIST criteria; or bidimensional, if based on the World Health Organization (WHO) measurements) or on the consideration of new lesions (specifically unmeasurable lesions) to define if there is progression or not; the criteria also differ in regard to incorporating new lesions in the baseline tumor burden (Table 2).

**Table 2:** Comparison of response evaluation criteria in solid tumors (RECIST), immune-related response criteria (irRC), immune-related RECIST (irRECIST), immune RECIST (iRECIST), and immune-modified RECIST (imRECIST)

	RECIST 1.1	irRC	irRECIST	iRECIST	imRECIST
Number of target lesions	Two per organ, 5 total	Five per organ, up to 10 visceral and 5 cutaneous	Two per organ, 5 total	Two per organ, 5 total	Two per organ, 5 total
Measurable size and dimension	Solid-organ lesions 10 mm in long axis, 15 mm in short axis for lymph nodes One-dimensional	≥ 5 × 5 mm Two-dimensional	Solid-organ lesions 10 mm in long axis, 15 mm in short axis for lymph nodes One-dimensional	Solid-organ lesions 10 mm in long axis, 15 mm in short axis for lymph nodes One-dimensional	Solid-organ lesions 10 mm in long axis, 15 mm in short axis for lymph nodes One-dimensional
Tumor burden	SLD of all target lesions	Sum of the products of the 2 greatest perpendicular dimensions of all target lesions	SLD of all target lesions	SLD of all target lesions	SLD of all target lesions
Unmeasurable lesions	1. Measurable lesions not selected as target lesions 2. Unmeasurable disease (including lesions too small to measure) 3. Other types of lesions that are difficult to measure in a reproducible manner^a^	Not specifically defined; nontarget lesions derived from irRC include: lymphangitic carcinomatosis, skin involvement in breast cancer, and abdominal masses that can be palpated but not measured	Follow definitions from RECIST 1.1	Follow definitions from RECIST 1.1	Follow definitions from RECIST 1.1
Appearance of new lesions	New measurable lesions constitute PD Unequivocal progression of new nonmeasurable lesions leads to PD	New measurable lesions do not constitute PD automatically; incorporated into TTB New nonmeasurable lesions do not define progression but preclude CR	New measurable lesions do not constitute PD automatically; incorporated into TMTB Only unequivocal progression of newnonmeasurable lesions leads to an overall response of irPD	New measurable lesions are assessed according to RECIST 1.1 guidelines but are recorded separately and not included in the SLD for target lesions identified at baseline. Results in iUPD; iCPD only achieved if additional new lesions are present at the subsequent time point or if there is an increase in the size of previous new lesions New nonmeasurable lesions are recorded but are not included in the tumor burden. Results in iUPD (requires confirmed increase in number or size at 4–8 weeks to assign iCPD)	New measurable lesions do not constitute PD automatically; incorporated into TTB New nonmeasurable lesions do not define progression but preclude CR
Response assessment	*CR* Complete resolution of nonnodal lesions and < 10 mm short-axis for lymph nodes	*CR* (irCR) Complete disappearance of all lesions; confirmation by repeat, consecutive assessment ≥ 4 weeks from first documentation	*CR* (irCR) Complete disappearance of all measurable and nonmeasurable lesions; lymph nodes must decrease to < 10 mm (short-axis); confirmation is not mandatory	*CR* (iCR) Resolution of all lesions, confirmed at ≥ 4 weeks	*CR* Complete disappearance of all lesions; confirmation by repeat, consecutive assessment ≥ 4 weeks from first documentation
	*PR * ≥ 30% decrease in tumor burden	*PR* (irPR) Decrease in tumor burden ≥ 50% relative to baseline; confirmation by repeat, consecutive assessment ≥ 4 weeks from first documentation	*PR* (irPR) Decrease ≥ 30% in TMTB relative to baseline; nontarget lesions are irNN; no unequivocal progression of new nonmeasurable lesions	*PR* (iPR) Decrease ≥ 30% in tumor burden compared with baseline in the absence of any new lesion or progression of nontarget lesions Injury	*PR* Decrease ≥ 30% in tumor burden compared with baseline in the absence of any new lesion or progression of nontarget lesions
	*SD* Does not meet criteria for CR / PR / PD	*SD* (irSD) Does not meet criteria for irCR or irPR in the absence of irPD	*SD* (irSD) Failure to meet criteria for irCR or irPR in the absence of irPD	*SD* (iSD) Neither PR or PD in the absence of any new lesion or progression of nontarget lesions	*SD* Neither PR or PD in the absence of any new lesion or progression of nontarget lesions
	*PD * ≥ 20% increase in the SLD and ≥ 5 mm absolute increase compared with nadir, unequivocal progression in nontarget lesions, and/or appearance of new lesions Confirmation of PD not required. Clinical status is not included in assessment	*PD (irPD)* Determined only on the basis of measurable disease. Negated by subsequent non-PD assessment ≥ 4 weeks from the date of first documentation (lack of confirmation) ≥ 25% increase in the sum of the products of the 2 greatest perpendicular dimensions compared with baseline / nadir	*PD (irPD) * ≥ 20% increase or ≥ 5 mm absolute increase in TMTB compared with nadir or irPD for nontarget or new nonmeasurable lesions; confirmation of PD is recommended a minimum of 4 weeks after the first irPD assessment	*PD* iUPD: Increase ≥ 20% in tumor burden from nadir, progression of nontarget lesions or new lesions ^b^. Clinical stability is considered when deciding whether treatment is continued after iUPD iCPD: Progressive disease is confirmed (iCPD) if the next imaging assessment (performed 4–8 weeks after iUPD) confirms additional new lesions, further increase in previous lesion size, or further increase in existing target or nontarget lesions from iUPD	*PD* Determined only on the basis of measurable disease. Negated by subsequent non-PD assessment ≥ 4 weeks from the date of first documentation (lack of confirmation). ≥ 20% increase in SLD (RECIST) compared with baseline / nadir

*CR* complete response, *CT* computed tomography, *FDG-PET* fluorodeoxyglucose positron emission tomography, *iCPD* immune-confirmed progressive disease, *iCR* immune complete response, *iPR* immune partial response, *irCR* immune-related complete response, *irNN* nonirCR and nonirPD, *irPR* immune-related partial response, *irSD* immune-related stable disease, *iUPD* immune-unconfirmed progressive disease, *iSD* immune stable disease, *MRI* magnetic resonance imaging, *PD* progressive disease, *SD* stable disease, *SLD* sum of longest diameters, *TTB* total tumor burden, *TMTB* total measured tumor burden, *PR* partial response, *irPD* immune-related progressive disease

^a^Include bone and leptomeningeal metastases, malignant ascites, pleural or pericardial effusions, inflammatory breast disease, lymphangitic carcinomatosis, ill-defined abdominal masses, skin lesions, etc.

^b^iUPD requires confirmation; progression is confirmed in the target lesion category if the next imaging assessment after iUPD (4–8 weeks later) confirms a further increase in the sum of measures of target disease from iUPD

The anatomic or radiologic immunotherapy response criteria include the following:

- RECIST 1.1: Version 1.1 of the “conventional” RECIST criteria introduced in 2009 are the most frequently used criteria for solid tumor treatment response assessment. These criteria are the only criteria that have been validated and accepted by the main regulatory agencies [35–38]. However, according to RECIST, increased tumor size or the appearance of new lesions in a cancer patient would invariably imply PD, which would result in an incorrect assessment of those patients with pseudoprogression, for instance.
- *Immune-related response criteria* (irRC): The irRC were introduced in 2008, and their main contribution was to incorporate new measurable lesions into baseline reference index lesions according to a new concept called “total tumor burden”. In addition, unlike the timeline of the classic criteria, disease progression should be confirmed within 4 weeks. Some limitations of the irRC are as follows: (1) Based on the WHO criteria, the irRC cannot be compared to the RECIST criteria because of the use of bidimensional-type measurements; (2) worse assessment of node involvement; and (3) possible overestimation of treatment efficacy, because they do not take into consideration unmeasurable new lesions [39–41].
- *Immune-related RECIST* (irRECIST): Developed in 2014 with the intention of aligning irRC with RECIST 1.1, irRECIST simplified immunotherapy response assessment and allowed comparison with other clinical trials that used RECIST criteria. According to the irRECIST criteria, new measurable lesions are added to baseline lesions in a concept called “total tumor burden measured”. Similar to the irRC criteria, every disease progression case should be confirmed radiologically within a 4-week period [42, 43].
- *Immune RECIST* (iRECIST): These criteria were derived from a consensus among the RECIST work group, pharmaceutical industries, and the main regulatory agencies to standardize and validate immunotherapy response criteria. The iRECIST would become a consensus guideline assuring consistent data design and collection that would facilitate the analysis and compared interpretation of immunotherapy-based clinical trials. Unlike the other criteria, new lesions were not incorporated into baseline lesions, and thus, they were registered separately. One of the key concepts of iRECIST is immune-unconfirmed PD (iUPD), a category including cases at the first sign of PD to be confirmed within the next 4–8 weeks [41, 44, 45].

- Immune-modified RECIST (imRECIST). imRECIST was described in 2018 and was inspired by the irRC criteria principles. New measurable lesions were incorporated into baseline target lesions (unlike in iRECIST). The imRECIST criteria relate patterns of response or progression to overall survival through indirect assessment criteria (such as progression-free survival) [46].

### Key points and recommendations

- Although the classic RECIST criteria remain a useful tool to assess response to immunotherapy in the clinical practice, sometimes they underestimate the benefit of immunotherapy. Specific immune-related response criteria must be considered to evaluate patients under treatment with immunotherapy.

### Metabolic response criteria

Given the limitations of conventional imaging techniques for the assessment of immunotherapy-treated patients, new functional and molecular imaging techniques were considered.

Metabolic response criteria for solid tumors were proposed, because tumor response to new targeted molecular therapies in the field of oncology might manifest as decreased 18F-FDG uptake without a marked reduction in tumor size (Table 3).

- European Organization for Research and Treatment of Cancer (EORTC): The first PET-based criteria were proposed in 1999 to assess metabolic response in solid tumors [47] and were the first criteria applied to assess metabolic response to immunotherapy [48]. A CR is defined as a resolution of FDG uptake within the tumor that is indistinguishable from the surrounding normal tissue. A > 25% increase in tumor standard uptake value (SUV) or the appearance of new ^18^F-FDG-avid lesions defines progression.
- PET Response Criteria in Solid Tumors (PERCIST): Wahl et al. [49] proposed new response criteria applying the average SUV corrected by lean body mass (SUL) within a 1-cm^3^ spherical volume of interest (SULpeak) [50]. More than 30% or 0.8-unit increases in SULpeak or new ^18^F-FDG-avid lesions indicated progression.CR was defined as ^18^F-FDG uptake of target lesions lower than that of the liver and indistinguishable from the background.
- PET/CT Criteria for Early Prediction of Response to Checkpoint Inhibitor Therapy (PECRIT): Choet et al. [51] analyzed different criteria in 20 advanced melanoma patients treated with checkpoint inhibitors. They defined new morpho-functional early response criteria combining the change in the sum of RECIST 1.1-based target lesion diameters and the PERCIST SULpeak change in the hottest lesion, with a 95% diagnostic accuracy (sensitivity 100%, specificity 93%). A very interesting aspect of these criteria is the capacity to predict clinical benefit at 4 months, defined as RECIST CR, PR or SD based on an increase > 15.5% in the SULpeak of the hottest lesion. Therefore, the combination of morphologic and metabolic information constitutes the main tool for immunotherapy response assessment.
- PET Response Evaluation Criteria for Immunotherapy (PERCIMT). A prospective study was conducted recently and included 41 metastatic melanoma patients treated with ipilimumab [52]. The authors defined clinical benefit as SD, PR and CR, and to predict outcome, they established optimal cut-off points for the number of new lesions according to their functional diameter measured in fused ^18^F-FDG PET/CT images. Neither maximum SUV (SUVmax) nor mean (SUVmean) changes during immunotherapy were correlated with clinical response, but they observed that a cut-off of four new lesions was predictive of treatment failure.
- Immunotherapy-modified PERCIST (imPERCIST): Described in 2019, these criteria do not necessarily consider new lesions as disease progression, as opposed to the PERCIST criteria. New lesions are included in ^18^F-FDG tumor uptake quantification, and progression is defined as an increase > 30% in the sum of the SULpeak of up to 5 lesions from baseline and follow-up PET with a maximum of 2 lesions per organ [53]. In this setting, Ito et al. [53] analyzed the relationship between tumor ^18^F-FDG uptake changes and survival in melanoma patients treated with ipilimumab according to both the PERCIST standard criteria and the new imPERCIST criteria. The authors concluded that PERCIST-based tumor response criteria correlated significantly with overall survival, and the new imPERCIST criteria improved this correlation as well as the prognostic value of ^18^F-FDG PET/CTcompared to the results obtained using PERCIST.

**Table 3:** Summary of the response criteria proposed for use with ^18^F-FDG PET

Response	EORTC	PERCIST 1.0	PECRIT	PERCIMT	imPERCIST
Complete response (CR)	Complete resolution of ^18^F-FDG uptake	Complete resolution of ^18^F-FDG-avid lesion uptake^a^	CB Disappearance of all lesions (TL and NTL); short axis of the nodes < 1 cm; no appearance of new lesions (RECIST1.1)	CB Complete resolution of ^18^F-FDG-avid lesion^b^ uptake; no appearance of new lesions	Complete resolution of ^18^F-FDG-avid lesion uptake^b^
Partial response (PR)	Reduction of tumor SUVs by 15–25% after 1 CT cycle, and > 25% after > 1 cycle of CT	Decrease in SULpeak of the LU^b^: Rd > 30%, Ad > 0.8 units	CB Decrease > 30% in the SLD of the TL (RECIST 1.1)	CB Complete resolution of uptake of some^18^ F-FDGavid lesion^b^; no appearance of new lesions	Decrease in SULpeak of TL^b^: Rd > 30%, Ad > 0.8 units
Stable disease (SD)	< 25% increase in SUV or < 15% decrease (without increasing the extent of tumor uptake or 20% of the greatest dimension)	Does not meet the other response criteria	Does not meet the other response criteria CB > 15.5% SULpeak of the LU nCB ≤ 15.5% SULpeak of the LU	CB Does not meet the other response criteria	Does not meet the other response criteria
Progressive disease (PD)	Increase in ^18^F-FDG uptake > 25%; increase in the extent of tumor uptake or 20% of the greatest dimension; appearance of new lesions	Increase in SULpeak > 30% (> 0.8 units); unequivocal progression of NTL; new hypermetabolic lesions	nCB Increase > 20% in the SLD in the TL, at least 5 mm; new lesions (RECIST 1.1)	nCB Appearance of ≥ 4 new lesions with a FD < 1 cm; ≥ 3 new lesions with a FD of > 1 cm; ≥ 2 new lesions with a FD > 1.5 cm	SULpeak increase > 30% (> 0.8 units); unequivocal progression of other lesions (NTL)

*Ad* absolute decrease, *CB* clinical benefit, *FD* functional diameter, *LU* lesion with greater uptake, *nCB* no clinical benefit, *NTL* non-target lesions, *Rd* relative decrease, *SLD *sum of longest diameters, *SUL *SUV corrected by lean body mass, *SUV* Standard uptake value, *TL* target lesion^18^F-FDG, 18-fluorine-fluorodeoxyglucose

^a^Following a typical tumor pattern, distinguishable from changes produced by treatment or infection

^b^Usually, the lesion chosen for the posttreatment study is the same as that chosen for the baseline study, but it may be another lesion that has higher uptake in the study performed after treatment (as long as this lesion was previously present in the baseline study, even if the lesion did not have the greatest uptake in the baseline study)

### Key points and recommendations

- FDG PET should be performed before immunotherapy (for evaluating the whole metabolic tumour burden), after 2–3 cycles of immunotherapy, and at the end of treatment (for response assessment). New PET-based criteria must be introduced in clinical practice. These criteria should be used instead of PET Response Criteria in Solid Tumors (PERCIST), which do not enable progression disease to be distinguished from the atypical response patterns to immunotherapy. imPERCIST criteria may improve the prediction of overall survival and the assessment of response in patients treated with immunotherapy, and PERCIMT or PECRIT may identify patients who achieve clinical benefit from immune treatment, defined as complete response, partial response or stable disease.

## Predictive and prognostic biomarkers

One of the main clinical concerns related to immunotherapy is the identification of biomarkers allowing for the selection of patients who would respond to the therapy or for the prediction of atypical responses such as pseudoprogression or hyperprogression. Different types of biomarkers have been proposed, including PD-L1 expression, microsatellite instability, tumor mutational burden, T lymphocyte tumor infiltration or imaging tests.

### Serum/tissue biomarkers

Immune checkpoint inhibitors constitute the main immunotherapy treatment in clinical practice. Some biomarkers have been proposed allowing the identification of patient groups who would benefit more from PD-1/PD-L1 inhibitors than from other therapeutic options. Without a doubt, the best-known biomarker is PD-L1 expression in tumor cells [54] or in immunologic system cells [55]. The usefulness of this marker is low in general, because we frequently observe patients who do not express PD-L1 and nevertheless respond to the treatment, and vice versa, and even in some studies, a greater response has been described in patients with low-PD-L1-expression tumor cells [56, 57]. However, more than 50% PD-L1 tumor cell expression in non small cell lung cancer patients predicts higher effectiveness of pembrolizumab as a first-line treatment compared to the effectiveness of chemotherapy. Furthermore, PD-L1 expression is required by regulatory agencies like EMA to prescribe atezolizumab (PD-L1 > 5%) or pembrolizumab (PD-L1 > 10%) as a first-line treatment for urothelial cancer patients who are not candidates for chemotherapy with cisplatinum.

Nevertheless, the most relevant biomarker is microsatellite instability (observed in patients with DNA repair pathway alterations) and predicts a very high response rate to PD-1 /PDL-1 inhibitors [58]. This is why regulatory agencies have approved pembrolizumab for treating patients with tumors showing microsatellite instability, independent of their histological type. Finally, high tumor mutational burden has been linked to the effectiveness of immunotherapy [59], but given the conflicting results among several studies this biomarker has not yet been implanted in the clinic nor authorized by regulatory agencies.

Regarding biomarkers for monitoring disease progression, we would like to note that the interleukin-8 serum level directly correlates with tumor burden and that variations in interleukin-8 during treatment with PD-1/PD-L1 inhibitors are associated with clinical response [60]. This type of biomarker could be useful for determining treatment effectiveness in conjunction with imaging tests, especially in cases of difficult response assessment, such as pseudoprogression.

### Key points and recommendations

- Treatment with PD-1 / PD-L1 blockade should be prescribed based on biomarker expression in the following clinical situations:
  - Metastatic non-small cell lung cancer and metastatic bladder cancer: monotherapy with immune checkpoints inhibitors is restricted to patients with positive expression of PD-L1 tumor cells (TPS) or a composite positive score (CPS).
  - Pembroliuzmab is approved by the FDA for the treatment of adult and pediatric patients with unresectable or metastatic, microsatellite instability-high (MSI-H) or mismatch repair deficient (dMMR) solid tumors that have progressed following prior treatment and who have no satisfactory alternative treatment options, and for the treatment of unresectable or metastatic MSI-H or dMMR colorectal cancer that has progressed following treatment with chemotherapy.

### Radiological biomarkers

Several radiological findings derived from conventional imaging techniques might predict or suggest a response to immunotherapy. Thus, regardless of the classic criterion of size reduction, the halo sign can identify treatment response in patients with pulmonary metastases of melanoma undergoing treatment with immunotherapy [61]. Lesion density changes in computerized tomography (CT) have been considered as response criteria for other types of therapies. In this setting, Gray et al. evaluated melanoma patients treated with interferon and antiangiogenic therapy based on the morphology, attenuation, size, and structure (MASS) criteria, showing that they could predict progression-free survival and overall survival. Finally, new contrast media, such as ultrasmall superparamagnetic ironoxide (USPIO), tend to accumulate in tumor-associated macrophages and might be used for assessing immune cell infiltration levels in the tumor to evaluate possible immunotherapy responses by magnetic resonance imaging (MRI) [62].

Artificial intelligence has improved the analysis of medical images [63]. Radiomics, a method that extracts multiple quantitative data points from medical images, would allow the development of imaging biomarkers. Some image findings have been correlated with PD-L1 or IDO1 expression [63] or with the degree of tumor-infiltrating lymphocytes [64–66]. Chen et al. [66], by combining clinical and radiomic data extracted from the texture and morphological analysis of magnetic resonance images with hepatic-specific contrasts, could predict lymphocytic tumor-infiltration levels in hepatocellular carcinomas, and they found that tumors with higher lymphocyte infiltration were more homogeneous. Several parameters obtained from radiomic analysis of CT images were also correlated with clinical evolution after anti-PD-1 or PD-L1 therapies in patients with lung cancer [65, 67–70] or head and neck cancer [71].

Radiomics analysis of tumor structure, texture and heterogeneity might facilitate the obtainment of more accurate information on tumor biology. Radiomics could also be useful for assessing atypical responses and toxicities associated with immunotherapy. In this way, Tunali et al. [72] developed clinical radiological models able to predict possible hyperprogression and define poorer survival rates in patients treated with immunotherapy, and Colen et al. [73] established a radiomics-based model to predict immunotherapy-induced pneumonitis.

Functional imaging techniques have rarely been used as immunotherapy biomarkers. Nevertheless, whole-body MRI with diffusion-weighted sequences has been used for assessing rituximab-treated lymphoma patients, evidencing that apparent diffusion coefficient changes (ADCs) may allow the differentiation of responding from nonresponding patients [74, 75] and confirm an early response.

### Metabolic and molecular imaging biomarkers

PET imaging techniques offer qualified imaging biomarkers that can be used not only as predictors of therapy response but also as prognostic factors. The most frequently used markers are SUV and new quantitative indexes, such as metabolic tumor volume (MTV) and total lesion glycolysis (TLG). Bastiannet et al. [76] correlated SUV in melanoma-infiltrated lymph nodes with prognosis in patients with stage III melanoma, showing a significantly higher 5-year rate with lower (41%) than with higher (24%) SUVmean. However, other authors could not confirm this correlation between glucose uptake in PET and survival.

MTV defines the tumor volume (in cm3) by pathologic 18F-FDG uptake, and TLG is calculated by multiplying MTV by SUVmean, which weights the volumetric burden and metabolic activity of tumors. These biomarkers have demonstrated prognostic value in different solid and hematologic neoplastic tumors. Ito et al. [77] evaluated a cohort of 142 consecutive melanoma patients treated with ipilimumab, and MTV was an independent prognostic factor for overall survival (*p* = 0.001). Thus, patients with higher MTV showed lower survival than patients with below-average MTV (10.8 months versus 26 months, respectively). On the other hand, the combination of metabolic biomarkers with clinical prognostic factors such as age, LDL and brain metastasis allowed patient stratification into significantly different overall survival ranges.

^18^F-FDG PET radiomic analysis of tumors might provide more accurate information on tumor biology. The most commonly used parameters are those expressing 18F-FDG tumor metabolic heterogeneity, which has been correlated with higher relapse rates and has shown prognostic value for different tumor types [78]. A retrospective study conducted with patients with metastatic melanoma showed great metabolic heterogeneity in the metastasis of each patient and among patients. A higher 18F-FDG uptake showed a relationship with the lactate dehydrogenase (LDH) values [79]. Finally, a recent study showed better clinical outcome in colon cancer patients with higher tumor sphericity and lower tumor volume and 18F-FDG heterogeneity on PET [80].

### Key points and recommendations

- Apart of different molecular markers (PD-L1 expression, microsatellite instability, and mismatch repair deficiency), there are emerging imaging-based biomarkers to predict and evaluate response to immunotherapy and the risk of immune-related adverse events. However, its usefulness in the clinical practice is still limited.

## Functional and molecular imaging advances for immunotherapy response assessment

In preclinical models, multiparametric MRI (including contrast T2-, ADC- and T1-weighted imaging) has been used in glioblastoma multiforme brain tumors for an early response assessment of CAR-T and natural killer (NK) cell therapy [81, 82]. Promising techniques such as 19F MRI might facilitate in vivo assessment of monocytes and macrophages. The clinical use of functional imaging techniques has been limited in the assessment of immunotherapy response. The value of MRI diffusion has been demonstrated in lymphoma patients [74, 75, 83, 84], and CT perfusion has been applied for response assessment of interferon 1-treated metastatic renal cancer and carcinoid tumors [85, 86].Functional imaging techniques might also facilitate the differentiation between tumors and inflammatory sites in glioblastoma patients treated with dendritic cell immunotherapy [87].

On the other hand, immunoPET with specific labeled antibodies has great potential when the therapy depends on tumor target expression in lesions and enables assessment of biomarker expression evolution over time. Most immunoPET studies are preclinical or in the framework of clinical trials. 68 Ga-Granzyme B PET quantification is a highly sensitive and specific early predictor of therapeutic efficacy of checkpoint inhibitor therapy [88]. In non-small cell lung cancer patients, immunoPET with 89Zr-nivolumab enabled the noninvasive quantification of PD-1/ PD-L1 tumor expression, demonstrating PD-L1 tumor expression heterogeneity between metastases of the same patient and among different patients [89]. In addition, PD-1 tumor expression was observed with 18F-BMS-986192 in patients with low PD-L1 expression, as determined by immunohistochemistry, most likely due to tumor PD-L1 expression heterogeneity. Therefore, immunoPET might identify patients despite low PD-L1 expression in biopsies, assessing tumor microenvironment changes induced by treatment with predictive and prognostic value. In a study conducted with 89Zr-atezolizumab in lung, bladder and breast cancer patients, PET uptake exhibited high predictive value concerning treatment response and a strong correlation with survival [90].

Despite the promising data obtained by functional and molecular imaging techniques, clinical studies are necessaryto confirm these preliminary findings.

### Key points and recommendations

- In the future, immunoPET imaging, which offers new radioactive tracers that target key immune pathways and cellular immune responses, should be used to improve patient´s stratification, to predict the efficacy of immunotherapy, and to assess tumor response.
- Functional imaging techniques, including quantitative parameters derived from diffusion-weighted or perfusion imaging, have demonstrated a limited role in the assessment of immunotherapy. Nowadays, their clinical use would not be recommended beyond the research field.

## Conclusions

Cancer immunotherapy has led to a significant breakthrough in patients’ outcomes. In this setting, both conventional imaging techniques and classic response criteria have shown limited applicability for the assessment of immunotherapy response. To overcome these limitations, there is a growing need to maximize our ability to image the biological features associated with the interactions between tumors and the immune system. The combination of morphological and functional and/or metabolic response criteria together with the emergence of radiomics offers new approaches for the development of prognostic and predictive imaging biomarkers for immunotherapy response assessment.
